# ^18^F-labeled PEGylated exendin-4 imaging noninvasively differentiates insulinoma from an accessory spleen: the first case report of [18F]FB(ePEG12)12-exendin-4 positron emission tomography/computed tomography for insulinoma

**DOI:** 10.3389/fendo.2023.1245573

**Published:** 2023-08-31

**Authors:** Kentaro Sakaki, Takaaki Murakami, Hiroyuki Fujimoto, Yoichi Shimizu, Kanae Kawai Miyake, Daisuke Otani, Sakura Kiyobayashi, Takuya Okada, Masakazu Fujimoto, Takuro Hakata, Ichiro Yamauchi, Kotaro Shimada, Hironori Shimizu, Kazuyuki Nagai, Yuji Nakamoto, Nobuya Inagaki

**Affiliations:** ^1^ Department of Diabetes, Endocrinology and Nutrition, Graduate School of Medicine, Kyoto University, Kyoto, Japan; ^2^ Radioisotope Research Center, Agency for Health, Safety and Environment, Kyoto University, Kyoto, Japan; ^3^ Department of Diagnostic Imaging and Nuclear Medicine, Graduate School of Medicine, Kyoto University, Kyoto, Japan; ^4^ Department of Diagnostic Pathology, Graduate School of Medicine, Kyoto University, Kyoto, Japan; ^5^ Division of Hepatobiliary Pancreatic Surgery and Transplantation, Department of Surgery, Graduate School of Medicine, Kyoto University, Kyoto, Japan; ^6^ Medical Research Institute Kitano Hospital PIIF Tazuke-kofukai, Osaka, Japan

**Keywords:** exendin-4, PET, insulinoma, GLP-1 receptor, β-cell imaging

## Abstract

**Background:**

Insulinomas are the most common functioning pancreatic neuroendocrine neoplasms, and these tumors induce hypoglycemia due to hyperinsulinemia. Hypoglycemia caused by insulinomas can cause seizures, coma or death due to the delayed diagnosis. The only curative treatment is surgical resection. To perform curative surgical resection of insulinomas, preoperative localization is crucial. However, localization of insulinomas is often challenging using conventional imaging methods such as computed tomography (CT) and magnetic resonance imaging. Although endoscopic ultrasound (EUS) fine-needle aspiration and selective arterial calcium stimulation test, which can reflect the endocrine character of the tumor, are performed in such cases, these modalities are invasive and require operator-dependent techniques. Additionally, somatostatin receptor (SSTR)-targeted imaging has a relatively low sensitivity for detecting insulinomas due to its low SSTR type 2 expression. Thus, there is an urgent need for developing a noninvasive diagnostic technique which is specific for detecting insulinomas. Consequently, glucagon-like peptide-1 receptor-targeted imaging has recently emerged and gained a wide interest. Recently, we have developed a novel ^18^F-labeled exendin-4-based probe conjugated with polyethylene glycol, [^18^F]FB(ePEG12)12-exendin-4 (^18^F-exendin-4), for positron emission tomography (PET) imaging. Here we report a case of insulinoma in which ^18^F-exendin-4 PET/CT noninvasively provided critical information for localization.

**Case description:**

This is a case of a 58-year-old male with symptomatic hypoglycemia for 10 years; however, a preoperative diagnosis of insulinoma was not established due to the difficulty in differentiating it from an accessory spleen using conventional imaging. Moreover, the patient requested to avoid invasive diagnostic procedures including EUS. ^18^F-exendin-4 PET/CT revealed significant uptakes in the pancreatic tail whereas no apparent uptakes were observed in the spleen; thus, curative laparoscopic enucleation of the pancreatic tail was performed. The diagnosis of insulinoma was confirmed *via* histopathological examination. This is the first case report of insulinoma diagnosed using ^18^F-exendin-4 PET/CT.

**Conclusion:**

In this case, PET information led to curative resection through enucleation of the pancreas. ^18^F-exendin-4 PET/CT may serve as a useful noninvasive clinical tool for insulinoma localization.

## 1 Introduction

Insulinomas are the most common functioning pancreatic neuroendocrine neoplasms, which cause hypoglycemia due to hyperinsulinemia (1–5). Insulinomas occur in 1-4 people per million in the general population (3, 5). Hypoglycemia caused by insulinomas presents various symptoms like behavioral changes, amnesia, palpitations, tremor, and diaphoresis (3, 5). The hypoglycemia may lead to seizures, loss of consciousness, coma, or death (3, 5, 6). Surgical resection is the only curative management for insulinoma, and an accurate and precise preoperative localization is essential to successfully perform minimally invasive surgery. However, since most insulinomas are <2 cm in diameter, occasionally, tumor localization through conventional imaging methods, such as computed tomography (CT) and magnetic resonance imaging (MRI), is rendered difficult (7–10). In such cases, endoscopic ultrasound fine-needle aspiration (EUS-FNA) and selective arterial calcium stimulation test (SACST) are performed as they reflect the endocrine character of the tumor (7–10). However, these tests are invasive and require operator-dependent techniques (7–10). Somatostatin receptor (SSTR)-targeted imaging approach can be another alternative; however, it has low sensitivity for detecting insulinomas compared to other pancreatic neuroendocrine neoplasms (11). Moreover, SSTR imaging does not directly reflect the endocrinological function of the tumor such as insulin secretory capacity. While surgical resection is the only curative treatment for insulinoma, there are cases where surgery has not been performed due to the difficulties in the diagnostic procedure, particularly in preoperative tumor localization (8),

Recently, glucagon-like peptide-1 receptor (GLP-1R)-targeted imaging has emerged as a noninvasive specific diagnostic tool for insulinomas (10, 12, 13). Furthermore, several positron emission tomography (PET) imaging methods using exendin-4-based probes such as gallium-68 (^68^Ga), copper-64, and zirconium-89-labeled exendin-4 derivatives have been developed (14). Particularly, in several clinical studies, ^68^Ga-labeled exendin-4 derivatives have been used and successfully detected insulinomas with high sensitivity (15, 16). However, some reports elucidated that PET/CT using ^68^Ga-labeled exendin-4 has some limitations; ^68^Ga has a short half-life and has a limited supply and distribution to a large number of hospitals. Moreover, it has strong renal uptake signals rendering it difficult to visualize lesions near the kidneys, such as those in the tail of the pancreas (14, 15, 17).

In recent years, owing to its low positron emission energy and potential for rendering high-resolution images, fluorine-18 (^18^F) is widely used in nuclear medicine; however, PET imaging of pancreatic β cells with ^18^F-labeled exendin-4 probes has not yet been employed in human clinical cases with insulinoma (12). We developed a novel GLP-1 R-targeted imaging method using a polyethylene glycol (PEG) conjugated (PEGylated) exendin-4-based probe, [^18^F]FB(ePEG12)12-exendin-4 (^18^F-exendin-4) PET/CT (18, 19). PEGylation has been demonstrated to improve the pharmacokinetic properties of various peptide analogues used in cancer diagnosis and therapy (18, 20). PEGylation can increase their hydrophilicity while reducing their sensitivity to proteolysis, thereby decreasing renal clearance and hepatic accumulation (20). ^18^F-exendin-4 PET/CT showed favorable sensitivity and specificity for insulinoma detection with mice transplanted with rat insulinoma cells (INS-1 cells), as well as in a disease model of mice with multiple simultaneous insulinomas (18). Moreover, the clinical safety of this probe has been demonstrated in healthy human subjects (21).

Here, we report the first clinical case of insulinoma in which ^18^F-exendin-4 PET/CT noninvasively provided tumor localization leading to a successful curative laparoscopic surgery with minimal invasion.

## 2 Case description

A 58-year-old man was referred to our hospital for symptomatic hypoglycemia for the past 10 years.

**Figure 1:**
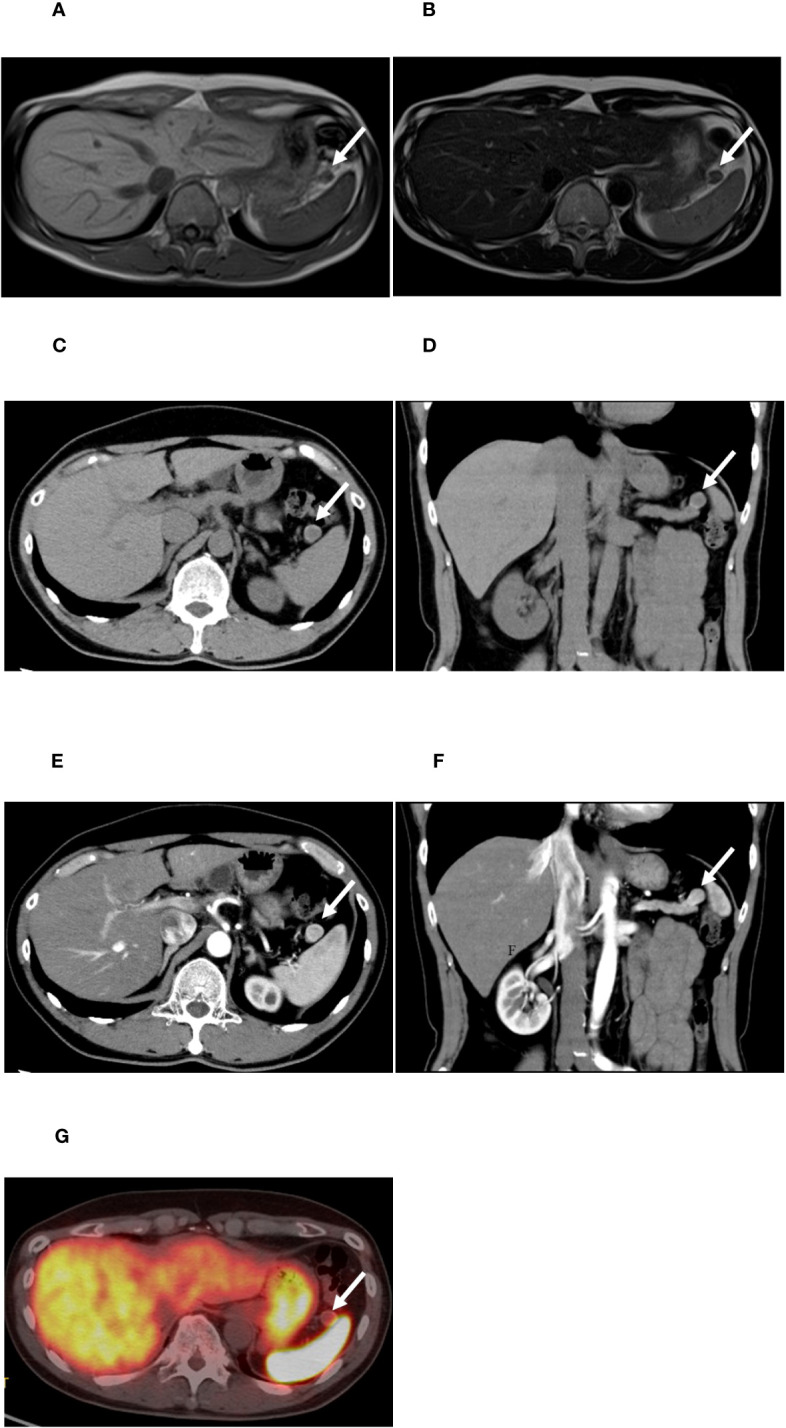
Images obtained by conventional clinical examinations. **(A, B)** Magnetic resonance imaging of the abdomen. **(A)** T1-weighted image. **(B)** T2-weighted image. **(C–F)** Computed tomography (CT) scan of the nodule of the pancreatic tail. **(C, D)** Plain CT; **(E, F)** early phase of contrast-enhanced CT. **(G)** ^68^Ga-labeled 1,4,7,10-tetraazacyclododecane-N,N’,N’’,N’’’-tetraacetic acid-d-Phe1-Tyr3-octreotide (DOTATOC) positron emission tomography (PET)/CT. The white arrows indicate the nodule in the tail of the pancreas.

At the age of 48, he manifested with pre-prandial malaise, discomfort, and amnesia. Since food or sugar intake resolved these symptoms, he did not seek for a medical consult. However, at the age of 49, a blood test revealed a low random serum glucose level (53 mg/dL), with a hemoglobin A1C (HbA1c) level of 4.7%. Subsequently, he underwent a contrast-enhanced CT scan at the age of 50, which revealed a 15-mm diameter nodule with intense early enhancement at the tail of the pancreas, which was adjacent to the spleen. The nodule was round in shape, and its enhancement effect was almost similar to that of the spleen. Abdominal MRI also showed a nodule in the same area. The signal was isointense with the spleen on T1-weighted imaging, while on T2-weighted imaging, the signal was almost equivalent or slightly hypointense compared to the spleen (**Figures 1A, B**). ^18^F-fluorodeoxyglucose PET/CT showed no significant uptake in the nodule. Since it was difficult to rule out the presence of an accessory spleen and establish the diagnosis of insulinoma, EUS and EUS-FNA were recommended as an additional preoperative investigation for further confirmation prior to surgery. However, the patient refused these imaging modalities due to its invasive nature. Since the symptoms of hypoglycemia could be managed with a supplementary diet, the patient was carefully followed up with dietary treatment as per his request. Thereafter, his random serum glucose levels remained at 47–90 mg/dL and his HbA1c levels remained at 4.6–5.0%. At the age of 58 years, his symptoms started to worsen. His regular blood sampling revealed that his random serum glucose level was 36 mg/dL and HbA1c was 4.5%, which were the lowest values on his clinical record during follow-up. Therefore, he was referred to our hospital and admitted for further examination.

His past medical history included hypertension, supraventricular tachycardia, and spinal disc herniation. His regular medications included cilnidipine and bisoprolol. He had no history of diabetes mellitus or other endocrinopathies and previous gastric surgery. Since the age of 40, he had no history of alcohol consumption and had not been using any anti-diabetic agents.

On admission, his blood pressure was 134/92 mmHg, and his heart rate was 63 beats per minute. His height was 171.0 cm and body weight was 73.9 kg. In the last 10 years, his body weight had increased by approximately 10 kg. Hyperinsulinemic hypoglycemia was confirmed in his fasting blood sample in the early morning (plasma glucose level was 44 mg/dL, serum insulin level was 5.1 μU/mL, C‐peptide level was 1.53 ng/mL, and total ketone body was 23.9 μmol/L; acetoacetic acid and 3-hydroxybutyric acid levels were 7.9 μmol/L and 16.0 μmol/L, respectively). His anti-insulin antibody was negative. His adrenocorticotropic hormone, cortisol, thyroid‐stimulating hormone, free thyroxine, and insulin-like growth factor 1 levels were within normal limits (14.7 pg/mL, 13.26 μg/dL, 1.664 μIU/mL, 1.52 ng/dL, and 181 ng/mL, respectively) (**Supplementary Table 1**).

Contrast-enhanced CT scan of the abdomen showed a well-demarcated hypervascular round nodule in the pancreas tail (**Figures 1C–F**), which was adjacent to the spleen. Compared to the CT and MRI images at the age of 50, no apparent change of the tumor size was noted. Findings suggestive of a lymph node or liver metastasis were not detected. For SSTR-targeted imaging, ^68^Ga-labeled 1,4,7,10-tetraazacyclododecane-*N,N*′,*N*″,*N*‴-tetraacetic acid-D-Phe^1^-Tyr^3^-octreotide (DOTATOC) PET/CT, which can provide higher spatial resolution and superior diagnostic capability than ^111^In-diethylenetriaminepentaacetic acid octreotide single-photon emission CT (SPECT)/CT, was performed (22). DOTATOC PET/CT showed no significant uptake in the pancreatic tail nodule (**Figure 1G**).

**Figure 2:**
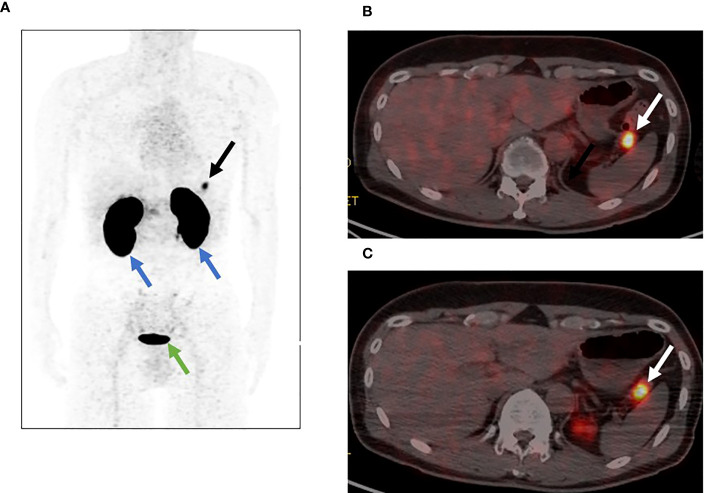
[^18^F]FB(ePEG12)12-exendin-4 PET/CT for the pancreatic tail nodule. The arrows indicate the nodule in the tail of the pancreas. **(A)** Maximum intensity projection of PET 1 h after administration. The black arrow indicates the nodule in the tail of the pancreas, the blue arrows indicate the kidneys, and the green arrow indicates the bladder. **(B)** Fusion imaging of PET/CT 1 h after administration. The white arrow indicates the nodule in the tail of the pancreas. **(C)** Fusion imaging of PET/CT 2 h after administration. The white arrow indicates the nodule in the tail of the pancreas.

^18^F-exendin-4 was synthesized according to the previous reports (18, 21) and ^18^F-exendin-4 PET/CT scans were performed. The protocol was approved by the Kyoto University Certified Review Board (Y0074) and was conducted in accordance to the provisions of the Declaration of Helsinki. ^18^F-exendin-4 PET/CT demonstrated distinct uptakes in the pancreatic nodule at both 1 and 2 h after the intravenous probe administration (**Figures 2A–C**). The maximum standardized uptake values (SUVmax) of the nodule were 14.5 at 1 h (**Figure 2B**) and 12.7 at 2 h (**Figure 2C**), respectively. These uptakes were remarkably higher than the normal pancreas or surrounding organs except for the kidney and bladder (**Figure 2A**). The nodule signals were easily distinguished from the normal pancreas and the surrounding organs including the spleen. The tumor/pancreas ratio of the mean standardized uptake values (SUVmean) were 9.31 and 9.28 at the corresponding time points. The SUVmean of the spleen were 0.62 and 0.35 at the corresponding time points. No other focal uptakes were observed in the whole body (**Figure 2A**). No adverse events were observed during the scans.

Additionally, an SACST was performed. SACST demonstrated greater than two-fold increases in insulin levels in the proximal and distal splenic arteries, without increases in the proper hepatic artery, gastroduodenal artery, or superior mesenteric artery (**Table 1**). These results suggested the presence of a responsible tumor in the tail of the pancreas, which was consistent with the findings of ^18^F-exendin-4 PET/CT.

**Table 1:** The results of the selective arterial calcium stimulation test (SACST).

	PHA	GDA	SMA	Proximal SpA	Distal SpA
Before load	14.7	13.1	17.9	16.5	19.1
20 seconds	14.8	12.0	17.1	43.4	78.5
40 seconds	15.5	14.4	15.5	459.8	249.5
60 seconds	15.1	19.0	12.7	826.2	501.8
90 seconds	13.1	14.8	12.9	952.1	593.2
120 seconds	12.2	10.7	11.7	800.0	432.0

Serum insulin levels (μU/mL) before and after the administration of calcium gluconate to each artery. PHA, proper hepatic artery; GDA, gastroduodenal artery; SMA, superior mesenteric artery; SpA, splenic artery.

**Figure 3:**
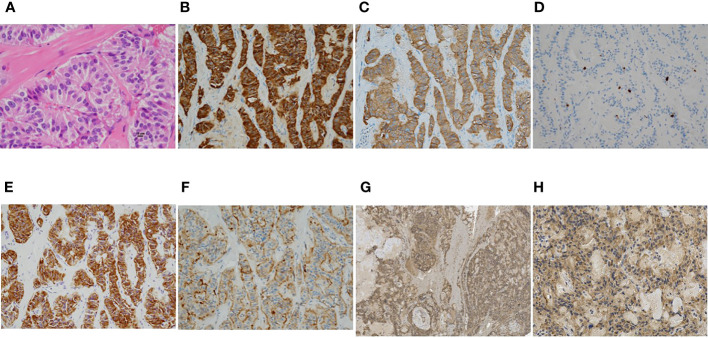
**(A–G)** Microscopic findings of the representative resected tumor. **(A)** Hematoxylin and eosin staining revealed a typical pancreatic neuroendocrine neoplasm, characterized by a trabecular proliferation of cells with oval nuclei and eosinophilic cytoplasm. **(B–G)** Immunohistochemical stains showed tumor cells positive for chromogranin A **(B)** and synaptophysin **(C)**. The Ki-67 labeling index was 2.0% **(D)**. The tumor cells were also positive for insulin **(E)**, somatostatin receptor 2 **(F)**, and glucagon-like peptide-1 receptor **(G, H)**. Magnification: ×400 **(A)**, ×200 **(B-F,H)**, ×40 **(G)**, scale bar: 10 μm **(A)**.

Therefore, the pancreatic tail nodule was diagnosed as insulinoma and was the responsible tumor for the symptomatic hypoglycemia. Based on the preclinical diagnosis, laparoscopic enucleation was performed. In the histopathological examination of the tumor, hematoxylin and eosin staining revealed a typical well-differentiated pancreatic neuroendocrine neoplasm, characterized by a trabecular proliferation of cells with oval nuclei and eosinophilic cytoplasm (**Figure 3A**). In immunohistochemistry, the cells of the nodule were positive for chromogranin A (**Figure 3B**) and synaptophysin (**Figure 3C**). The Ki-67 labeling index was 2.0% (**Figure 3D**). The cells of the nodule were also positive for insulin (**Figure 3E**), SSTR type 2 (**Figure 3F**), and GLP-1R (**Figures 3G, H**). **Figure 3G** (low magnification image) indicates that GLP-1R is diffusely expressed in the tumorous component. **Figure 3H** (high magnification image) indicates that GLP-1R is expressed in almost all cells. Based on these pathological findings, the tumor was confirmed as a well-differentiated, benign insulinoma, classified as NET G1 under the 2019 WHO classification (23).

After surgery, the hypoglycemic episodes and hyperinsulinemia resolved. The patient’s fasting plasma glucose levels returned to normal (fasting plasma glucose level, 98 mg/dL; insulin level, 5.7 μU/mL; and c-peptide level, 1.24 ng/mL) and his HbA1c level was 6.1%. Three months after surgery, no recurrence of hypoglycemia was observed.

## 3 Discussion

Ideal probe targets specific to β cells have been explored for clinical pancreatic β-cell imaging such as PET and SPECT (12). GLP-1R is considerably expressed in β cells, whereas its expression level in other endocrine cells has been reported as absent or low (24); furthermore, GLP-1R expression in pancreatic exocrine cells is also low (25). Importantly, GLP-1R is highly expressed in human insulinomas, whose expression levels were obviously higher than normal pancreatic β cells (12, 26, 27). Accordingly, GLP-1R is considered as the most promising and suitable target not only for clinical pancreatic β-cell imaging but also for insulinomas (8, 12). In particular, owing to the biological peptide stability of exendin-4 and the spatial resolution of PET, radiolabeled GLP-1R agonist exendin-4-based probes for PET imaging have been developed. Although some exendin-4-based probes for SPECT and PET imaging exhibited their utility in clinical studies (11, 12, 15, 16, 19, 28, 29), exendin-4-based probes with various chemical modifications are still being investigated for further improvement (12).

Although hyperinsulinemic hypoglycemia was manifested by the patient suggesting the existence of an insulinoma, localization of the responsible tumor was clinically difficult through conventional imaging modalities. Although a nodule was identified in the tail of the pancreas, the differential diagnosis between insulinoma and accessory spleen remained an obstacle for the curative surgery. Since the patient strongly refused any invasive procedures for tumor localization, ^18^F-exendin-4 PET/CT was considered as a suitable option to provide further conclusive information to establish the preoperative diagnosis. ^18^F-exendin-4 PET/CT showed significant uptakes in the pancreatic nodule and preoperatively localized the insulinoma noninvasively, which contributed to a successful minimally invasive surgery, leading to ultimate cure. In the current clinical setting, localization of insulinoma remains challenging even with invasive and operator-dependent techniques; thus, ^18^F-exendin-4 PET/CT may be a useful noninvasive insulinoma-specific imaging method, although the sensitivity and specificity for detecting insulinoma should be further investigated in a large-scale clinical trial.

In the present case, the responsible tumor was located in the tail of the pancreas and was very close to the left kidney. One of the potential limitations of ^68^Ga-labeled exendin-4 probes could be the increased tendency of probe accumulation in the kidney, which may hamper visualization of the tumors in the pancreatic tail near the kidney (17). Fortunately, our PEGylated probe ^18^F-exendin-4 was reported to show improved tumor uptake and demonstrate a higher contrast with the surrounding organs including the kidneys since PEGylation is known to improve pharmacokinetics and probe delivery to the targeted tumors which is attributed to its ability to increase the molecular weight of the probe and its stability in circulation. Moreover, ^18^F provides low positron emission energy and has a potential for rendering high-resolution images (18). Since the tumor in this case could be clearly visualized and substantially discriminated from the signal of the kidney with a reasonable distance, ^18^F-exendin-4 might be useful for the detection of insulinoma located in the pancreatic tail. Further investigation is warranted to establish the clinical sensitivity of ^18^F-exendin-4 PET/CT for insulinomas located in the pancreatic tail, including the influence of the probe signals from the kidneys.

An accessory spleen is a congenital abnormality consisting of a normal splenic tissue located separately from the anatomical location of the spleen. An accessory spleen is observed in approximately 10%–30% of the general population (30) and is commonly located in the pancreas. Intrapancreatic accessory spleens are typically round and under 2 cm in diameter and often show a characteristic homogenous contrast-enhanced appearance on CT scans with distinct borders. Distinguishing between an accessory spleen and pancreatic endocrine neoplasms including insulinoma occasionally becomes a clinical issue in establishing a differential diagnosis, as their CT, MRI, and ultrasound images show quite similar findings as these present as hypervascular nodular lesions (31). As for SSTR-targeted imaging, both can show probe uptakes and provide no additional information in the differential diagnosis since both an accessory spleen and pancreatic endocrine neoplasms express SSTRs (32, 33). Conversely, GLP-1 R is specifically expressed in pancreatic β cells, not in the spleen or splenic tissues (34). Thus, exendin-4 PET/CT can be considered to distinguish between an accessory spleen and insulinoma. In the present case, ^18^F-exendin-4 PET/CT provided a clear visualization of the insulinoma but did not reveal a significant uptake in the spleen, which confirmed the final diagnosis.

In this clinical case, ^18^F-exendin-4 specifically accumulated in the insulinoma as well as the pancreas, except for the excretory routes of the probe such as the kidneys and bladder. This was consistent with the findings of the phase 1 clinical study of the probe (21) and the organ distributions of GLP-1R expression (26). Moreover, in the present case, the probe uptake in endogenous pancreatic β cells did not disturb the delineation of insulinoma on the image analysis since the probe uptake in the insulinoma was much higher than the normal pancreas, which was consistent with the previous preclinical findings (18).

It is reported that almost all the benign insulinomas express high levels of GLP-1R, whereas the malignant insulinomas tend to express less GLP-1R (26). The insulinoma in the present case was well-differentiated benign tumor and was clearly visualized by ^18^F-exendin-4 PET/CT. Although there is a previous report that (111)In-[Lys(40)(Ahx-diethylenetriaminepentaacetic acid [DTPA])NH (2)]-exendin-4 SPECT/CT visualized 4 out of 11 cases of malignant insulinomas (35), ^18^F-exendin-4 PET/CT might hold the potential for better detection in malignant insulinomas since our probe is PEGylated to enhance its delivery efficiency to the tumor and PET can provide higher spatial resolution (18). Further investigations such as a large-scale clinical study including malignant insulinomas should be warranted.

## 4 Conclusion

We reported the first case of insulinoma diagnosed using a novel GLP-1R targeted imaging, ^18^F-labeled PEGylated exendin-4 PET/CT. In this case, information provided by PET, which was conducted noninvasively, resulted in curative resection through enucleation of the pancreas instead of partial pancreatic resection. ^18^F-exendin-4 PET/CT may well be a useful tool to localize insulinomas.

## Data availability statement

The original contributions presented in the study are included in the article/**Supplementary Material**. Further inquiries can be directed to the corresponding authors.

## Ethics statement

This protocol of 18F-exendin-4 PET/CT was approved by the institutional review boards of Kyoto University (Y0074) and was registered in the Japan Registry of Clinical Trials (Registration No. jRCTs051200156, https://jrct.niph.go.jp/latest-detail/jRCTs051200156). It conformed to the provisions of the Declaration of Helsinki. Written informed consent was obtained from the individual for the publication of any potentially identifiable images or data included in this article.

## Author contributions

KSa and TM contributed to the conception and design of the study and wrote the manuscript. TM also contributed to data analysis and discussions, and reviewed and edited the manuscript. HF, DO, and SK contributed to data analysis, data collections and discussions. YS, KM, and YN synthesized the probe and performed imaging tests. KSh and HS performed the selective arterial calcium stimulation test. KN performed surgical treatment. TH and IY took responsibility for patient management. TO and MF performed the histological diagnosis of the tumor. NI contributed to overall relevant discussions and reviewed the manuscript. All authors contributed to the article and approved the submitted version.
