# Supplementary material for: 18F-labeled PEGylated exendin-4 imaging noninvasively differentiates insulinoma from an accessory spleen: the first case report of [18F]FB(ePEG12)12-exendin-4 positron emission tomography/computed tomography for insulinoma
Source: Front Endocrinol (Lausanne). 2023 Aug 31;14:1245573. doi: 10.3389/fendo.2023.1245573 (PMC10501723; doi:10.3389/fendo.2023.1245573)
Supplement: Supplementary file 1 [file Table_1.docx]

# Supplementary Table 1.

The result of fasting blood test in the early morning.

| Plasma glucose level | 44 mg/dL |
| --- | --- |
| Serum insulin level | 5.1 μU/mL |
| Serum C-peptide | 1.53 ng/mL |
| Total ketone body | 23.9 μmol/L |
| Acetoacetic acid | 7.9 μmol/L |
| 3-hydroxybutyric acid | 16.0 μmol/L |
| Anti-insulin antibody | < 125 nU/mL |
| Adrenocorticotropic hormone | 14.7 pg/mL |
| Cortisol | 13.26 μg/dL |
| Thyroid‐stimulating hormone | 1.664 μIU/mL |
| Thyroxine | 1.52 ng/dL |
| Insulin-like growth factor 1 | 181 ng/mL |
